# Needs assessment for patient-centered education and outcome metrics in robotic surgery

**DOI:** 10.1007/s00464-022-09500-7

**Published:** 2022-08-24

**Authors:** Hope Nicole Moore, Thais Reif de Paula, Deborah S. Keller

**Affiliations:** 1 Department of Surgery, Davis Medical Center, University of California, 2335, Stockton Blvd, NAOB 6Th Floor, Sacramento, CA 95817 USA; 2 Biomedical Sciences Department, University of Houston Medical School, Houston, TX USA

**Keywords:** Robotic surgery, Robotic-assisted surgery (RAS), Patient experience, Informed consent, Patient related experience metrics (PREM)

## Abstract

**Background:**

From clinical experience, many patients undergoing robotic assisted surgery (RAS) have a poor understanding of the technology. To ensure informed consent and appropriate expectations, a needs assessment for patient-centered education and outcome metrics in RAS is warranted. Our goal was to perform an assessment of patient understanding, comfort with robotic technology, and ability to obtain critical information from their surgeon when undergoing RAS.

**Methods:**

Twenty patients planned for RAS by three surgeons were asked to complete a six-item Likert agreement scale survey prior to signing informed consent. The study coordinator administered surveys, while the surgeon left the room. Indicator statements were crafted to reduce bias and two-way evaluated for consistency. The surgeons were additionally asked their perception of each patient’s understanding and comfort with RAS. Frequency statistics and tendencies were analyzed.

**Results:**

Surgeons strongly agreed all patients appropriately understood how RAS functioned and would ask more questions before signing consent, if needed. Patients were predominately not familiar with RAS and felt surgeons did not explain how RAS worked. There was wide variability on if patients understood how RAS worked for their treatment. Overall, patients were not completely comfortable with RAS for their care, did not understand the risks of RAS compared to other approaches, and did not feel their surgeon understood what they needed to know to make informed decisions.

**Conclusions:**

This needs assessment demonstrated critical gaps in patient knowledge about RAS, surgeon communication skills, and the ability of surgeons to know what was important from the patient perspective. The development of RAS patient-centered education and outcome metrics could help address these gaps.

The use of robotics in general surgery has grown exponentially over the past decade worldwide [1]. Since 2012, utilization of robotic-assisted surgery (RAS) increased dramatically across all procedures, and certain operations shifted by an order of magnitude towards greater robotics use [2]. Colorectal is one subspecialty with particularly strong growth, as the robot’s technology facilitates a high-definition view for meticulous dissection and greater flexibility maneuvering in the deep pelvis [3–5]. There are concerns that the growth of RAS outpaces the evidence to support its use and additional costs [2]. Implementation is further driven by competition in health care markets [6]. With the increasing demand and rapid implementation of RAS, there has been a lack of metrics on benefits, especially from the patient’s point of view.

There is increasing recognition of the need to involve patients in the processes of healthcare delivery and quality improvement [7]. The patient experience directly impacts surgical quality and is associated better safety and outcomes [8]. Patient-reported experience measures (PREMs) are tools that capture the overall patient experience of healthcare. Patient experience tools may be used to monitor patient feedback, the clinical effectiveness of care, and the general experience of a patient, rather than an experience related to a specific disease [9] These tools have strong positive associations between patient satisfaction and safety [10] and are reliable measures of how well a hospital can provide high quality service from a patient’s perspective [9]. Currently, no PREMs exist to help prioritize care or ensure appropriate communication for shared decision making in surgery [11]. Furthermore, none exist related to RAS. From the authors’ clinical experience, many patients undergoing RAS have a poor understanding of the technology. To ensure truly informed consent and appropriate patient expectations with robotic procedures, we believed a needs assessment for patient comprehension and comfort with RAS was warranted; to support our clinical suspicions, a feasibility study was planned as a preliminary step to determine if further study on patient’s understanding, acceptance, and ability to obtain information needed for informed consent could and should be done.

The goal of this study was to perform a feasibility study with a needs assessment of patient understanding, comfort with robotic technology, and ability to obtain critical information from their surgeon when planned to undergo RAS. We hypothesized that patients did not fully understand RAS or feel comfortable obtaining critical information from their surgeon- even when signing consent. We also hypothesized that surgeons may be unaware of patients’ knowledge gaps and inability to ask critical questions.

## Methodology and materials

A systematic needs assessment was performed with the objective to study the state of patient knowledge and comprehension about robotic assisted surgery. The target audience was patients undergoing colorectal surgery at an urban quaternary referral center, with the results planned to be given to the surgeons (stakeholders) performing informed consent and the surgery. Patients were included if over 18 years old, were planned to undergo an elective surgical procedure via an abdominal approach using RAS by a member of the Colorectal Surgery Division, and were deemed appropriate to proceed by the surgeon. Patients were excluded if less than 18 years old, unable to read or understand the study protocol and informed consent, were undergoing surgery through a planned open, laparoscopic, or endoscopic approach, or had previously had a robotic surgical procedure.

Twenty consecutive patients planned for robotic assisted colorectal resection were recruited from practice of three experienced colorectal surgeons. The sample size needed for validity from returned surveys in the entire target population at a confidence interval of 95% was 79. For the needs assessment, a 25% random sample was surveyed for validation [12]. To minimize biases, each surgeon was experienced in consenting patients and had performed over 100 robotic procedures at the time of the study.

Data was collected directly from the patients using a standardized survey instrument. The instrument was developed by a multidisciplinary team of patients, surgeons, and a social scientist using the end user acceptance, attitudes, and preference themes from the technology assessment model [13] and Universal Theory of Acceptance and Use of Technology model [14]. A consensus was reached on the initial patient survey questions by all parties after 3 rounds of review, where all agreed they were succinct, effective to highlight issues and help determine what type of education is needed to address the issues. Indicator statements were crafted to reduce bias and two-way evaluated for consistency. The questions were not open ended; a six-item Likert agreement scale was used to capture responses. The survey had concise instructions provided, and all questions were constructed in similar formats, organized around groups of similar questions, and had a consistent array of response categories. The final Patient Survey Questions are seen in Table 1. Surgeon stakeholders were also queried on their perception of the patient comprehension and ability to obtain more information about RAS from their surgeon (Table 1).

**Table 1:** Patient and surgeon survey questions

*Patient Survey Questions*
I was familiar with robotic assisted surgery prior this surgical consultation
My doctor or other member of my treatment team has explained exactly how robotic assisted surgery works for my treatment to me
I understand how robotic assisted surgery works for my treatment
I am completely comfortable with robotic assisted surgery technology for my care
My surgeon understands the information I need to make decisions about my surgery
I understand how robotic assisted surgery and its risks differ from other surgical approaches
*Surgeon Survey Questions*
The patient appropriately understands how robotic assisted surgery works and its risks and benefits compared to other options for their case
The patients would ask more questions before signing informed consent if needed

The patients were asked to complete the survey prior to signing informed consent during their pre-operative clinic appointment. At the time of the survey, the surgeons had fully explained their treatment plan and solicited questions. A study coordinator administered all surveys, while the surgeon stepped out of the room. After the patient exited the consultation room, the study coordinator surveyed the surgeons on that particular patient’s understanding of RAS and their ability to ask critical questions. The same study coordinator dispensed all surveys and performed the analysis.

From the survey results, frequency statistics and tendencies were analyzed. Spearman’s Rho was performed to measure the correlation between the patient and surgeon responses. The main outcome measures were the gaps in understanding and communication with RAS.

The study received Institutional Review Approval (#1768507-1). The Standards for Reporting Qualitative Research list for complete, transparent reporting was followed for the study protocol.

## Results

Twenty consecutive patients were recruited over a 3-week period in November and December 2020. All 20 patients recruited completed the survey in its entirety. The patient sample had a mean age of 53.62 (SD 14.28) and was 55% female (*n* = 11). The mean BMI was 29.38 kg/m2 (SD 7.33) and 4 patients were frail (20%). Six patients (*n* = 30%) had prior abdominal surgery; no patient previously had RAS. The sample was diverse in race. The majority of patients (85%, *n* = 17) had English as their primary language. The main indication for surgery was colorectal cancer (45%, *n* = 9). The procedures performed were 4 low anterior resections, 5 sigmoid colectomies, 4 ventral mesh rectopexy, 3 ileocolic resections, and 4 right hemicolectomies. Full demographic details of the patient sample are seen in Table 2.

**Table 2:** Patient demographic data

Sample Size	20
Mean Age (SD)	53.62 (14.28)
*Gender (n, %)*	
Female/Male	11 (55%)/ 9 (45%)
*Race (n, %)*	
Caucasian	11 (55%)
Hispanic	6 (30%)
Asian	2 (10%)
African American	1 (5%)
*Consent Language*	
English	17 (85%)
Spanish	3 (15%)
American Society of Anesthesiologists Score (Median, Range)	3 (1–4)
Mean Body Mass Index (SD)	29.38 (7.33)
Prior Abdominal Surgery	6 (30%)
Diabetic requiring medication	5 (25%)
Hypertension requiring medication	8 (40%)
Steroids within 30 days of surgery	2 (10%)
Current Tobacco Smoker	3 (15%)
Frail (mFI-5 of 0.6 and above)	4 (20%)
*Indication for Surgery*	
Colorectal Cancer	9 (45%)
Inflammatory Bowel Disease	5 (25%)
Diverticulitis	3 (15%)
Prolapse	3 (15%)

**Fig. 1:**
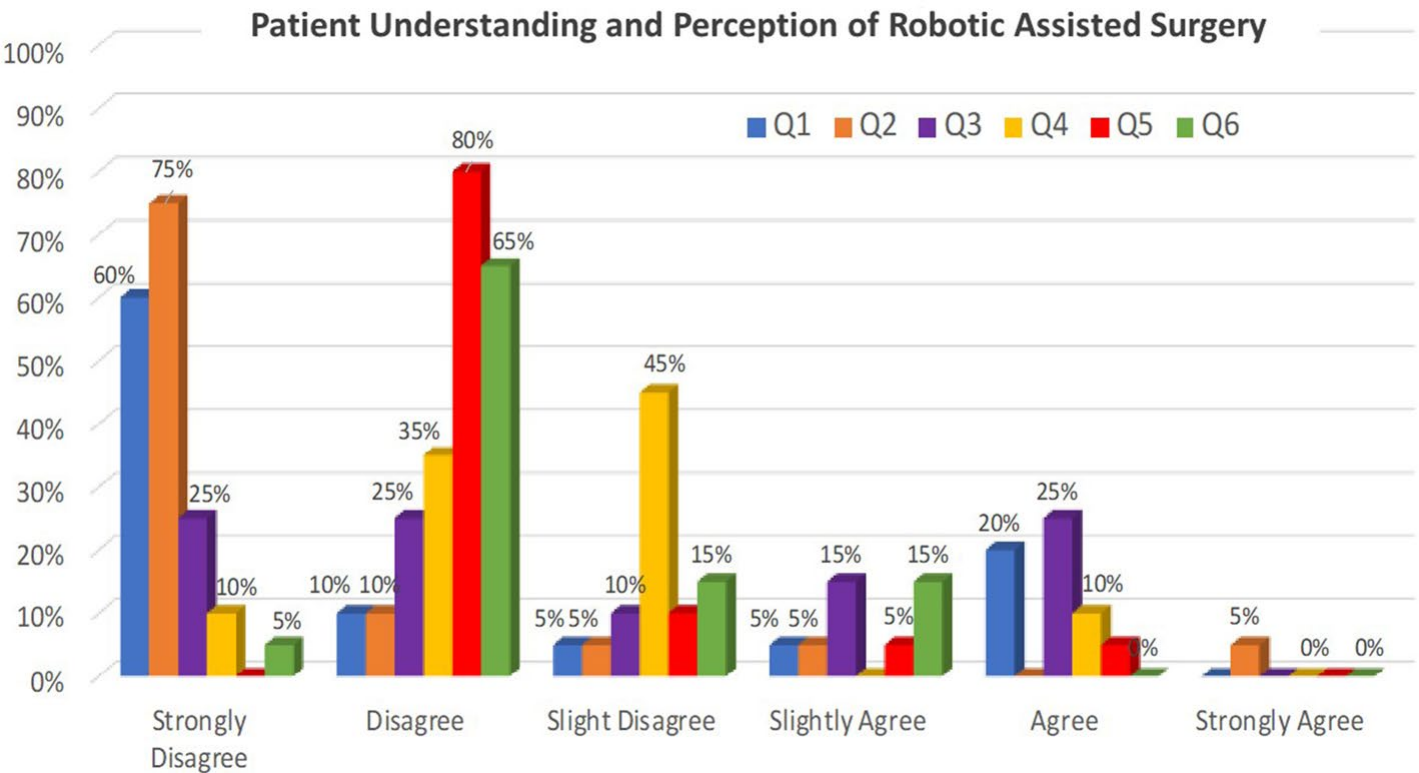
Patient understanding, perception, and comfort with robotic assisted surgery

From the patient surveys, 75% of patients were not familiar with robotic-assisted surgery prior this surgical consultation. Ninety percent of patients disagreed that their doctor or other member of the treatment team explained exactly how robotic assisted surgery worked for their treatment, with 75% strongly disagreeing. Only 25% of patients agreed that they understood how robotic assisted surgery worked for their treatment. Even fewer patients (15%) agreed that they understood how robotic assisted surgery and its risks differ from other surgical approaches. Just 15% agreed they were completely comfortable with robotic assisted surgery technology for their care. When asked if their surgeon understands the information they need to make decisions about their surgery, 90% of patients disagreed. The full response distribution from the patient surveys is seen in Fig. 1.

**Fig. 2:**
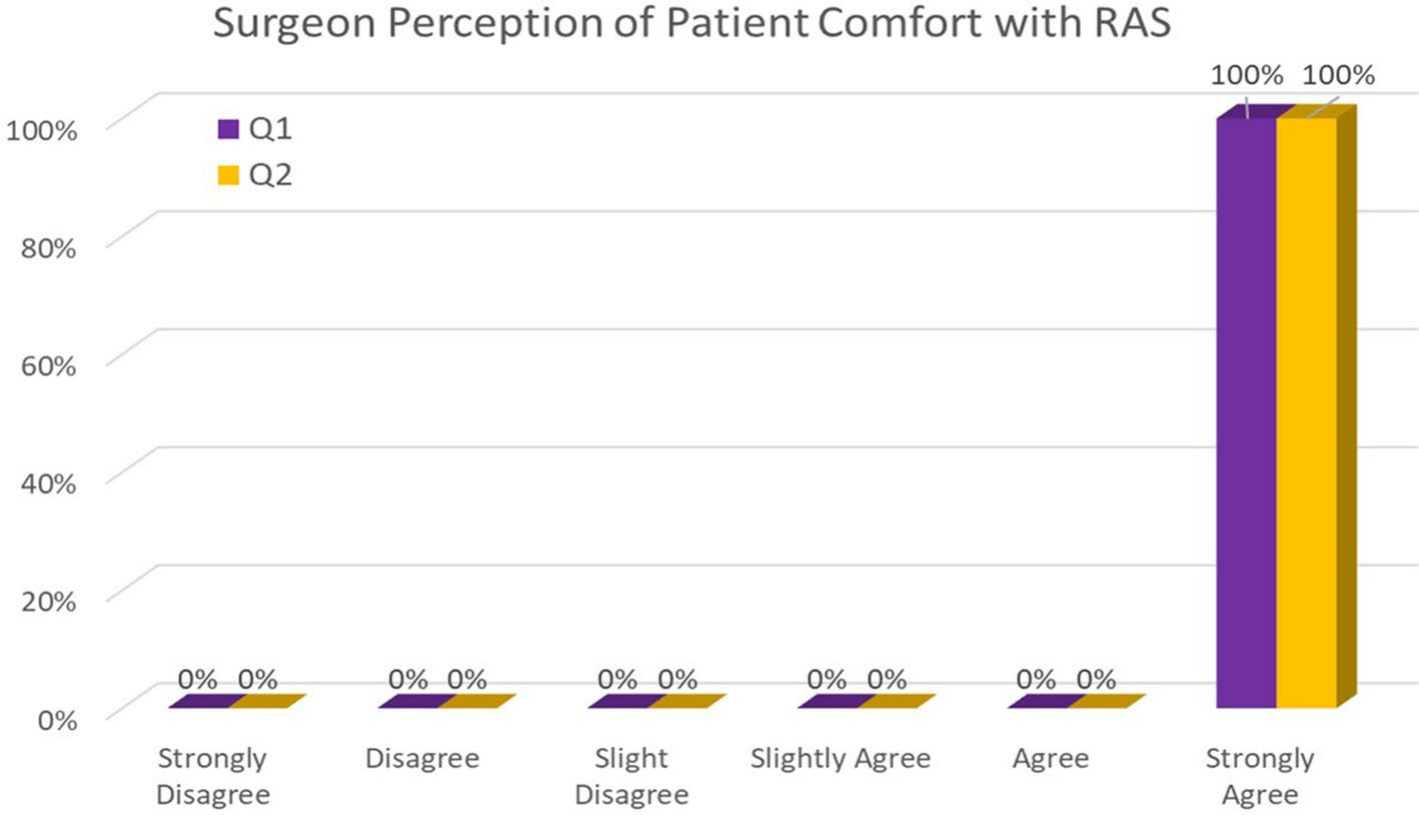
Relationship between patient and surgeon agreement on robotic-assisted surgery

All surgeons strongly agreed that all 20 patients appropriately understood how RAS works and its risks and benefits compared to other options. They also strongly agreed all patients would ask more questions before signing informed consent, if needed (Fig. 2).

**Fig. 3:**
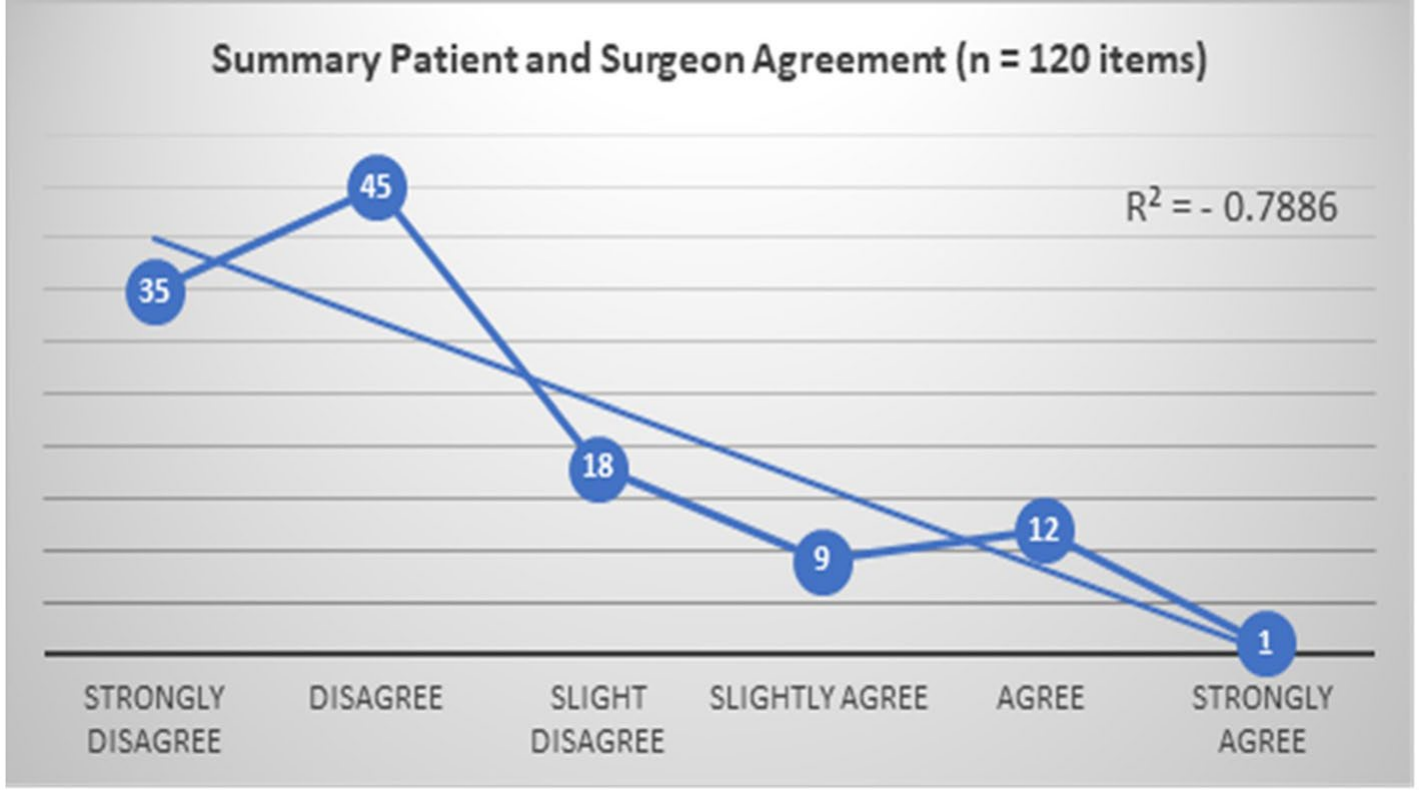
Relationship between patient and surgeon perception of RAS

In assessing the agreement between patient and surgeon responses, there was a very strong negative relationship between patient and surgeon perceptions for RAS with a Spearman’s Rho correlation of *p* = −0.8 (Fig. 3).

## Discussion

In this study, critical gaps were identified from the patient’s perspective on RAS, and the surgeon’s perception of their patient’s comprehension. Patients were predominately not familiar with RAS, did not understood how RAS worked for their treatment, or how its risks differed from other platforms. Patients did not feel that their surgeon explained RAS details well or understood what they needed to know about RAS to make informed decisions. It is clear that patients were not completely comfortable with RAS for their care. However, surgeons felt their patients had the appropriate knowledge and were able and willing to obtain details, if needed. This disparity highlights a need to assess the education and communication between the patient and surgeon relating to RAS.

Prior work has surveyed the public’s perceptions of RAS, with general results similar to our feasibility study results. In 2014, Boys et al. used a public crowdsourcing marketplace for the public’s perceptions about RAS, hospitals that have robots, and surgeons that use them [15]. Most respondents (86%) had heard of RAS and 72% felt RAS was safer, faster, less painful, and offered better results. Over half answered hospitals with a robot were better hospitals. However, 25% did not understand how RAS differed from open, laser, or scarless surgery and over 20% thought the robot had some degree of autonomy [15]. Fifty-five percent would prefer conventional laparoscopy over RAS. Recently, an online survey was performed by Mauddi et al. of 362 respondents that examined the public's perception of robotic surgery. They found respondents feared outcomes after RAS more than laparoscopic surgery (78.2% vs 14.9%, *p* < 0.001), and preferred laparoscopy to RAS (64.4% vs 35.6%, *p* < 0.001) [16]. In orthopedics specifically, a 30-question survey was completed by 588 people using a public crowdsourcing marketplace regarding RAS and patient engagement [17]. Respondents believe RAS led to better results (69%), fewer complications (69%), less pain (59%), and faster recovery (62%) than conventional methods. They also felt robotic surgeons were more skilled. However, respondents were concerned about lack of surgeon experience with RAS, harm caused by robot malfunction, and its increased cost. Only half accurately understood the robot’s role during surgery [17]. This lack of fundamental understanding could contribute to lack of informed patient decisions. However, these surveys were not targeted to patients; they were offered to the general public where respondents could be biased.

Query of patient knowledge and attitudes toward surgical approaches has been performed in gynecology, but not specifically pertaining to robotics. Irani et al. anonymously surveyed 219 patients seeking obstetrics and gynecology care at one medical center [18]. The authors found almost half of participants do not understand the difference between laparoscopic and robotic procedures, and over 2/3 did not know that the surgeon moves the robot's arms to perform the surgery. Thus, providers cannot assume patients have an adequate understanding of surgical options.

This work is the first survey of patient perceptions of RAS and their ability to obtain critical information from their surgeon as well as the first that assesses the relationship between the surgeon’s perception of patient comprehension and ability to obtain more information. We performed this preliminary work to query if there was truly an issue that needs to be further investigated and addressed. The results were striking. Aligned with prior work, most patients were not familiar with RAS and did not have a good understanding of how the robot worked. The majority did not feel their surgeon explained well how RAS worked for their treatment, did not understand the risks of RAS compared to other platforms, and did not have the information needed for consent. Most patients were not completely comfortable with RAS for their care. This survey was done at a time when consent would have been sought. A unique finding of this work was surveying the awareness surgeons had about their patient’s knowledge and comfort seeking information about their procedure. There was very strong disagreement between the parties on an understanding of RAS, its risks and benefits, and the ability to obtain critical information for their care. This is necessary for successful communication and ensuring truly informed consent for surgery. While the exact amount of information necessary to obtain informed consent is undefined, valid consent requires the patient to have received sufficient information to make a decision [19]. Here, the information routinely provided did not always translate to sufficient understanding. This demonstrates a need to improve the process.

We recognize the limitations of this work. The timing of the patient survey could provoke anxiety that interferes with the planned course of care. There was also a small sample size and consecutive patients used, which could introduce bias. However, we took care to craft statements that were not leading and to use a standardized objective for data collection and analysis. Though one center was used, several surgeons were included for generalizability. Though patient-surgeon communication disconnect likely exists with all platforms, in this work specific for robotic surgery the comments demonstrated confusion on if the robot or the surgeon was performing the surgery and the safety of the platform; these comments are unique to robotics, suggest a rudimentary lack of understanding even after a full consent conversation, and warrant specific action for RAS.

The important clinical implication from this work is the need to perform a full-scale prospective study into the patient perceptions of RAS. We confirmed that a study can be done and should be done, as well as informing on the best design. To this end, we followed up this feasibility study with a powered prospective structured needs assessment of the patient perceptions of RAS and priorities for surgery and recovery. The data collection is complete, and analysis is underway. These results can be used to develop meaningful PROMS and PREMS to confirm appropriate comprehension, comfort, and ability to obtain information about RAS for the best patient experience. These focused tools are needed as the most applicable instrument currently available is the Hospital Care Quality Information from the Consumer Perspective survey (HCAHPS). However, the lack of relationship between patients' perspectives of care from HCAHPS and the incidence of 30-day postoperative morbidity and mortality within a statewide surgical collaborative call to question the use of HCAHPS scores to inform patient decision-making and quality improvement [20]. Results can also be used to help develop effective communication tools for surgeons and patients regarding RAS.

## Conclusions

This study demonstrated critical gaps in patient knowledge and comfort with RAS, patient and surgeon communication skills, and the ability of surgeons to perceive what was important from the patient perspective. To ensure informed consent and appropriate patient expectations with robotic procedures, a full needs assessment to develop relevant patient-centered education and outcome metrics in RAS is warranted. A paradigm shift in surgical care towards shared decision making could also help address the discrepancy between the patient perceptions of robotic surgery and the clinical reality perceived by the surgical team. Future studies will assess the impact of these initiative on surgical quality.
